# Internal Transcribed Spacer 2 (nu ITS2 rRNA) Sequence-Structure Phylogenetics: Towards an Automated Reconstruction of the Green Algal Tree of Life

**DOI:** 10.1371/journal.pone.0016931

**Published:** 2011-02-10

**Authors:** Mark A. Buchheim, Alexander Keller, Christian Koetschan, Frank Förster, Benjamin Merget, Matthias Wolf

**Affiliations:** 1 Department of Biological Science and the Mervin Bovaird Institute for Molecular Biology and Biotechnology, The University of Tulsa, Tulsa, Oklahoma, United States of America; 2 Department of Bioinformatics, Biocenter, University of Würzburg, Am Hubland, Würzburg, Germany; J. Craig Venter Institute, United States of America

## Abstract

**Background:**

Chloroplast-encoded genes (*mat*K and *rbc*L) have been formally proposed for use in DNA barcoding efforts targeting embryophytes. Extending such a protocol to chlorophytan green algae, though, is fraught with problems including non homology (*mat*K) and heterogeneity that prevents the creation of a universal PCR toolkit (*rbc*L). Some have advocated the use of the nuclear-encoded, internal transcribed spacer two (ITS2) as an alternative to the traditional chloroplast markers. However, the ITS2 is broadly perceived to be insufficiently conserved or to be confounded by introgression or biparental inheritance patterns, precluding its broad use in phylogenetic reconstruction or as a DNA barcode. A growing body of evidence has shown that simultaneous analysis of nucleotide data with secondary structure information can overcome at least some of the limitations of ITS2. The goal of this investigation was to assess the feasibility of an automated, sequence-structure approach for analysis of IT2 data from a large sampling of phylum Chlorophyta.

**Methodology/Principal Findings:**

Sequences and secondary structures from 591 chlorophycean, 741 trebouxiophycean and 938 ulvophycean algae, all obtained from the ITS2 Database, were aligned using a sequence structure-specific scoring matrix. Phylogenetic relationships were reconstructed by Profile Neighbor-Joining coupled with a sequence structure-specific, general time reversible substitution model. Results from analyses of the ITS2 data were robust at multiple nodes and showed considerable congruence with results from published phylogenetic analyses.

**Conclusions/Significance:**

Our observations on the power of automated, sequence-structure analyses of ITS2 to reconstruct phylum-level phylogenies of the green algae validate this approach to assessing diversity for large sets of chlorophytan taxa. Moreover, our results indicate that objections to the use of ITS2 for DNA barcoding should be weighed against the utility of an automated, data analysis approach with demonstrated power to reconstruct evolutionary patterns for highly divergent lineages.

## Introduction

Researchers for a host of organisms have turned to DNA barcoding as a powerful, new tool in the study of diversity. Although the literature is replete with cautionary statements regarding DNA barcoding [1], [2], [3], [4], [5], [6], a large number of studies have suggested that the benefits of barcoding either outweigh the problems or that most problems can be addressed [7], [8], [9], [10], [11], [12], [13], [14], [15], [16].

Much of our own research interests have focused less on the issue of species delimitation but rather more on the phylogenetics of chlorophytan green algae [17], [18], [19], [20], [21], [22], [23], [24], [25], [26], [27], [28], [29], [30], [31], [32]. Nonetheless, our own work [17], [18], and the work of many others [36], [37], [38], [39], [40], [41], [42] have revealed the utility of the nu ITS2 rRNA (ITS2) gene in studies of closely related green algae. It has become abundantly clear that much of the data gathered in our purely phylogenetics efforts have tremendous potential for use in DNA barcoding for the Chlorophyta.

Barcoding efforts within the Viridiplantae (green plants) have, as one might expect, largely focused on vascular plants, in general, and flowering plants, in particular [8], [14], [43], [44], [45], [46], [47], [48], [49], [50]. Genomic targets for potential land plant barcodes have included chloroplast (*rbc*L, *atp*B, *mat*K, *psb*A, *rpo*C1, *rpo*B, *ndh*J, *acc*D), mitochondrial (COX [CO]1) and nuclear genes (various single copy genes, ITS1, ITS2, 5.8S) [51]. Chen et al. (2010) concluded that many of these potential markers are inappropriate for barcoding due to low variability (e.g., *rpo*B, *ndh*J, *acc*D, *atp*B, COX1, 5.8S rRNA) or suffer from difficulties in amplification (e.g., ITS1 rRNA and nuclear, single copy genes). The chloroplast encoded *mat*K gene (with *rbc*L) has been formally selected as a DNA barcoding candidate for the land plants [50]. However, the absence of *mat*K from all green algae except the charophytes [52], [53], [54] renders moot, the question of its utility for the Chlorophyta.

It remains possible that one or more of the problematic genomic targets noted above could be useful for studies of chlorophytan barcoding. However, at present, only the 5.8S rRNA and ITS1 rRNA genes have been studied in more than fifty chlorophytan taxa (3025 GenBank citations). Moreover, if the goal is to identify and test a universal (at least for the Viridiplantae) barcoding candidate, it is important to target only those candidates that will be of use for the land plants. Of those potentially suitable genomic targets that remain, only the cp *rbc*L (2477 current GenBank citations) and nu ITS2 rRNA (3418 current GenBank citations) genes have been routinely targeted for assessing chlorophytan diversity. Investigations of the *rbc*L gene from Chlorophyta have failed to identify a set of universal primers that successfully yield amplicons for all Chlorophyta [17], [55], [56], [57], [58]. Moreover, attempts to obtain *rbc*L data from cladophoralean green algae (Ulvophyceae) have largely been unsuccessful (only 3 GenBank citations as of 10/10/2010). Because of the extreme heterogeneity in *rbc*L across the green algae, the *rbc*L is, effectively, a non-universal gene. In contrast, the nu ITS2 gene from virtually all Viridiplantae can be amplified with a single set of universal primers [59]. Despite a relatively short length (128–483 bases across the Chlorophyta), some have even suggested that the nu ITS2 rRNA may be useful for comparisons within much of the domain Eukarya [60], [61], [62], [63], [64]. On the basis of the efficiency of amplification, the nu ITS2 rRNA gene is preferable to the cp *rbc*L. In addition, as a nuclear gene, the nu ITS2 rRNA gene is likely to have broader taxonomic applicability (i.e., beyond Viridiplantae) should it be deemed a good DNA barcode.

Many of the limitations first associated with the nu ITS2 rRNA (e.g., too much variation, too few nucleotide sites) have been overcome by secondary structure analysis which has systematically identified regions of variability as well as areas of substantial conservation [61], [62], [64], [65], [66], [67]. Furthermore, a simulation study recently confirmed the benefit of a sequence-structure approach [68]. Analyses of the simulated data resulted in the most robust trees, as assessed by the bootstrap, when secondary structure data were included in the phylogenetic reconstruction [68]. Moreover, the addition of sequence-structure permits the comparison of a much broader phylogenetic spectrum [68]. In reinforcing the conclusions from the simulation study, recent sequence-structure analyses of ITS2 data from lepidopterans permitted alignment of a broad taxonomic spectrum and yielded phylogenetic reconstructions that matched the resolution provided by analyses of COI and COII [69].

Much of the progress in establishing a nu ITS2 rRNA tool for diversity assessment, has been accomplished as a consequence of new bioinformatics applications, concepts and resources [35], [64], [65], [67], [70], [71], . In particular, the ITS2 Database III has substantially advanced the effectiveness of phylogenetic analyses using ITS2 data. At present, the ITS2 Database III, mined from the NCBI database, comprises over 250,000 structures (both partial and complete) that covers the range of eukaryotic diversity [73]. One of the innovations that is coupled with the database is the use of Hidden Markov Models to more fully automate the annotation pipeline [73]. The final stage of the pipeline involves homology-modelling that provides the user with a sequence-structure assessment that is the product of a phylogenetically broad, comparative approach [73]. Given the bioinformatics support coupled with the relative ease of obtaining comparable data, the nu ITS2 rRNA appears to be a superior candidate for use in phylogenetic reconstruction of large data arrays and as a DNA barcode for the Chlorophyta.

One goal of this study is to evaluate the use of an automated workflow that includes those analyses suggested by Schultz and Wolf [66] and that can be accomplished within a reasonable time frame on an ordinary desktop computer. The need for automated procedures without further manual corrections in phylogenetics and species delineation is obvious, as the number of available sequences on public databases grows daily.

A secondary goal of this investigation is, however, a demonstration of the potential utility of the nu ITS2 rRNA as a DNA barcode for the Chlorophyta as tested against phylogenetic assessments based on other markers. The green algal class, Chlorophyceae, in particular, has been the target of numerous phylogenetic investigations in which the nu ITS2 rRNA gene was included as a genomic target [17], [18], [29], [32], [36], [39], [41], [76], [77], [78], [79], [80], [81], [82], [83]. These chlorophycean investigations, which represent only a portion of the total body of work in which the nu ITS2 rRNA gene has been used to study chlorophytan diversity (>80 published manuscripts), clearly show the utility of this marker in addressing species level questions. Our challenge is to determine if the use of automated analytical methods with both primary and secondary structural analysis yield robust trees that are largely congruent with other data (e.g., 18S rRNA, 26S rRNA, *rbc*L, *atp*B).

As part of the current investigation, we completed a pilot investigation of the potential for the ITS2 to serve as a DNA barcode for the class Chlorophyceae, which we, then, extended to include the whole of the phylum, Chlorophyta. Results from our tests of this approach clearly indicate that the nu ITS2 rRNA data possess considerable power to reconstruct reasonably robust hypotheses that are congruent with past work that employed markers that have been deemed “more conservative” than the nu ITS2 rRNA gene. Our results indicate that ITS2 has the potential to serve as a powerful tool for phylogenetics in an extraordinarily broad taxonomic context that may eventually encompass virtually the entirety of the domain Eukarya. Furthermore, the empirical results of our investigation suggest that the general antipathy to the implementation of ITS2 as a DNA barcode may not be wholly warranted.

## Results

**Figure 1:**
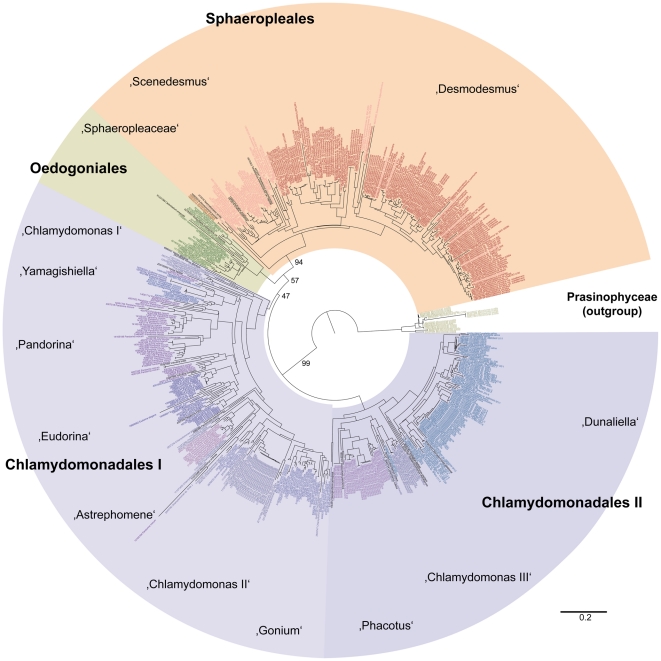
PNJ tree (with bootstrap values from 100 replicates) for sequence-structure data from the nu ITS2 rRNA gene for a comprehensive sampling of the class Chlorophyceae. Major taxonomic groups are labelled and highlighted using differential color coding.

The aligned nu ITS2 rRNA data for the class Chlorophyceae yielded a tree (Fig. 1) that resolved data representing the orders Oedogoniales (*Oedogonium*, *Bulbochaete* and *Oedocladium*), Sphaeropleales (*Desmodesmus*, *Scenedesmus*, *Atractomorpha* and *Sphaeroplea*), and Chlamydomonadales/Volvocales (*Chlamydomonas* [three non-monophyletic clades], *Yamagishiella*, *Pandorina*, *Eudorina*, *Astrephomene*, *Gonium*, *Phacotus* and *Dunaliella*). Two distinct chlamydomonad alliances were resolved (with only weak bootstrap support) by the ITS2 data (Fig. 1). The Sphaeropleales were resolved as monophyletic with high bootstrap support (94%). Furthermore, distinct lineages corresponding to putative chlorophycean species are preserved by the analytical protocol utilized in this experiment (Fig. 1).

**Figure 2 pone-0016931-g002:**
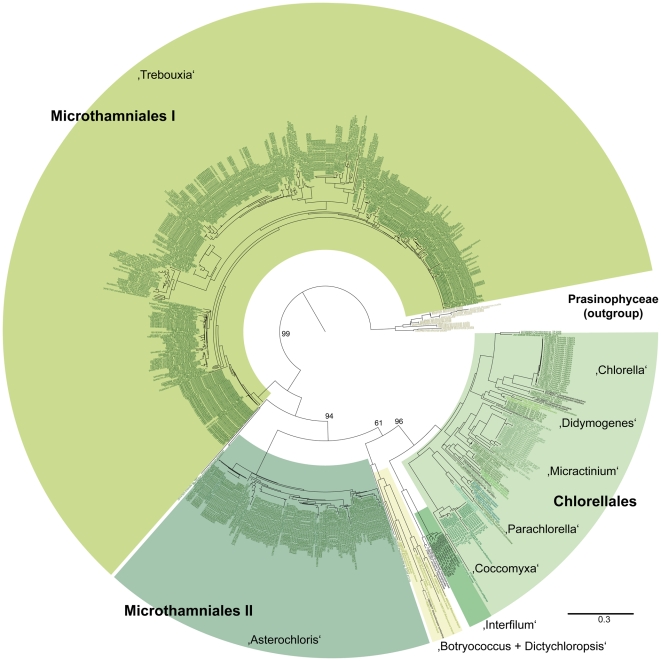
PNJ tree (with bootstrap values from 100 replicates) for sequence-structure data from the nu ITS2 rRNA gene for a comprehensive sampling of the class Trebouxiophyceae. Major taxonomic groups are labelled and highlighted using differential color coding.

**Figure 3 pone-0016931-g003:**
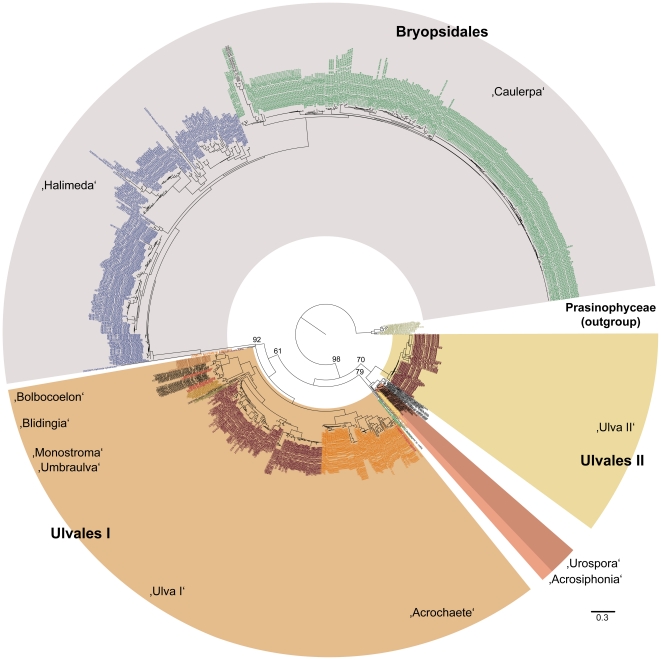
PNJ tree (with bootstrap values from 100 replicates) for sequence-structure data from the nu ITS2 rRNA gene for a comprehensive sampling of the class Ulvophyceae. Major taxonomic groups are labelled and highlighted using differential color coding.

Given the success of the experiment with data from the Chlorophyceae, the test was extended to include a comprehensive sampling of nu ITS2 rRNA sequence data from the green algal classes, Trebouxiophyceae (741 sequences) and Ulvophyceae (938 sequences). These data were analyzed under the same analytical conditions as the Chlorophyceae, including the use of prasinophycean data as the outgroup. The PNJ analysis resolved three principal clades of trebouxiophycean taxa (Fig. 2) that correspond to two sets of microthamnialean taxa (the *Trebouxia* alliance [Microthamniales I] and the *Asterochloris* alliance [Microthamniales II) and the Chlorellales which includes *Chlorella*, *Parachlorella*, *Coccomyxa*, *Micractinium* and *Didymogenes*. Bootstrap values for these three clades are 99%, 94% and 96%, respectively. Results of a third PNJ analysis (Fig. 3) revealed high bootstrap support for a Bryopsidales clade (92% bootstrap support; *Halimeda* and *Caulerpa* alliances). A *Urospora*/*Acrosiphonia* clade was resolved with 79% bootstrap support. Neither of the two ulvalean alliances (Ulvales I: *Bolbocoelon*, *Blidingia*, *Monostroma, Umbraulva* and one group of *Ulva* taxa; Ulvales II: a second group of *Ulva* taxa) were robustly resolved. However, the Ulvales II clade formed a sister group with the *Urospora*/*Acrosiphonia* alliance with 70% bootstrap support. As with the chlorophycean data (Fig. 1), the trebouxiophycean (Fig. 2) and ulvophycean (Fig. 3) data revealed numerous distinct branches that correspond to putative species.

**Figure 4:**
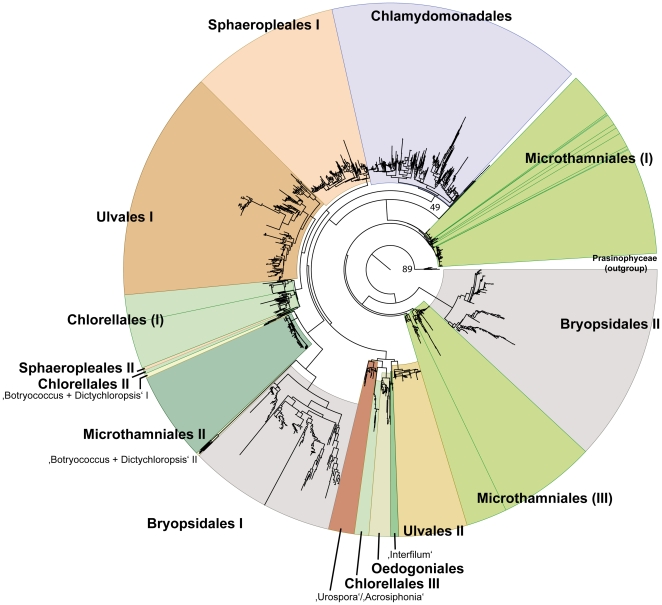
PNJ tree for sequence-structure data from the nu ITS2 rRNA gene for a comprehensive sampling of the phylum Chlorophyta. Major taxonomic groups are labelled and highlighted using differential color coding.

A composite, phylum-level analysis of ITS2 data (Fig. 4) derived from each of the class-level analyses reveals the same major clades for each class of green algae. However, the branching order of some of these clades differs between class-level and phylum-level analyses. The class level analyses, by default, present each class as monophyletic (Figs. 1–3). In contrast, the phylum level analysis challenges, albeit weakly, the monophyly of each of the classes (Fig. 4). For the Chlorophyceae, the Oedogoniales are allied with Ulvales I and Chlorellales III (*Coccomyxa*), a subset of the Sphaeropleales (Sphaeropleales II [Sphaeropleaceae]) are allied with Chlorellales I (*Chlorella*, *Parachlorella*, *Micractinium*, *Didymogenes*, *Diacanthos*, *Closteriopsis*, *Actinastrum*, *Dictyosphaerium*, *Auxenochlorella*, *Lobosphaeropsis*), II (*Pseudochlorella*, *Koliella*), and Microthamniales II (Fig. 4), and Sphaeropleales I (*Desmodesmus* and *Scenedesmus*) is sister to Ulvales I. The Chlamydomonadales are resolved as a monophyletic sister group to the latter alliance (Fig. 4). The Trebouxiophyceae form four distinct, non-monophyletic clades comprising the Microthamniales I, Microthamniales II, Chlorellales III, and Microthamniales II + Chlorellales I + Chlorellales II (Fig. 4). The Ulvophyceae also form four, non-monophyletic clades comprising the Bryopsidales II (*Caulerpa*), Ulvales + *Urospora*/*Acrosiphonia*, Bryopsidales I (*Halimeda*), and Ulvales I (Fig. 4).

Results from ML analyses of sequence data only (Supplemental files S1, S2, S3, and S4) indicate that the ML approach and the sequence-structure approach using NJ (Figs. 1–4) are topologically congruent in resolving the same major groups of taxa in each of the three class-level analyses and in the phylum-level analysis. However, the relative positions of a number of these groups differ in comparisons of the two distinct analyses for each of the four taxon sets (Chlorophyceae, Trebouxiophyceae, Ulvophyceae and Chlorophyta).

## Discussion

The independent analyses for each chlorophytan class generally recover phylogenetic signal that is consistent with studies of 18S rRNA [17], [18], [19], [25], [26], [27], [28], [29], [30], [31], [32], [41], [84], [85], [86], [87], 26S rRNA [18], [19], [25], [88], [89], *rbc*L [17], [55], [56], [58], [90], [91], [92], [93], [94] and *atp*B [17], [55], [56], [93].

Topological differences do exist between results with ITS2 data and other data sets. For example, analyses of the ITS2 data for the Chlorophyceae place the Chlamydomonadales as a basal, paraphyletic assemblage in the class (Fig. 1), whereas, both 18S and 26S rRNA data place the Oedogoniales, Chaetophorales and/or Chaetopeltidales as basal members of the class [19], [25]. However, these differences can be attributed to (1) weak support in one or both sets of data, (2) substantial differences in taxon sampling (e.g., no ITS2 data for Chaetopeltidales or Chaetophorales are available), (3) substantial differences in outgroup rooting, or (4) some combination of these influences. In addition to differences between phylogenetic results from ITS2 and other data sets, differences between results from class-level and phylum-level analyses of ITS2 data were also observed. For example, the class level analysis challenges the monophyly of Chlamydomonadales (Fig. 1), but the phylum level analysis (Fig. 4) resolves the order as monophyletic. Again, these differences are not robust and, thus, can be attributed to weak support, taxon sampling error or both.

The similarities and differences between the results from a NJ analysis using sequence-structure data (Figs. 1–4) and a ML analysis using sequence data alone (Supplemental files S1, S2, S3, and S4) are difficult to interpret given that it is not possible to discriminate between the effects of the model, the method, and the influence of the secondary structure data. Nonetheless, one or more of these factors are influencing the outcome of phylogenetic reconstruction. These observations highlight the need to expand the sequence-structure approach to include character-based methods of tree-building (e.g., ML). A union of sequence-structure analysis with character-based tree-building methods will create new opportunities for hypothesis testing that have the potential to further enhance the use of a sequence-structure approach in standard phylogenetic analyses, as well as, for DNA barcoding.

Our results represent further evidence that the ITS2 data can be aligned for a taxonomically broad set of organisms and that the alignment yields corroborated alliances of chlorophytan taxa. Most importantly, our results confirm that the analytic procedure does not lead to a loss of signal for the resolution of discrete, species level branches. The behavior of the ITS2 in conjunction with the automated, secondary-structure-based alignment compels us to conclude that the ITS2 data can be used to reconstruct chlorophytan phylogeny. As such, ITS2 has the potential to be a good choice for DNA barcoding in the Chlorophyta.

The remarkable results for the ITS2 gene from chlorophytan taxa raise the question: can these data and analytical approaches be applied to other organisms? Given that ITS2 data already exist for so many disparate groups of organisms, there is little doubt that this protocol could be easily extended to other members of the domain Eukarya. Recent work, which validates the use of ITS2 in barcoding embryophyte plants and animals, strongly supports this assertion [95]. As with most tools, there will be situations that may negate the utility of the ITS2 for phylogenetic analysis or as a DNA barcode. For example, some parasitic taxa have been identified as possessing substantially shortened ITS2 genes [96]. The ability of the analytical method to recover data from shortened sequences has yet to be tested in a broad taxonomic context.

One of the more problematic issues for the use of ITS2 for phylogenetic reconstruction or as a DNA barcode is that of heterogeneity. As part of the rDNA array, multiple, homogeneous copies of the ITS2 are presumed to exist within all eukaryotic organisms (ironically, making it an excellent barcode candidate due to greater ease of amplification). An assumption of homogeneity, as a consequence of concerted evolution [97], [98], may be unrealistic for a number of organisms [99], including at least some chlorophytes [42], [100]. Since heterogeneity of the rDNA array is an issue for the use of ITS2 in an ordinary phylogenetic analysis [101], the problem is not merely a product of its use in DNA barcoding. Consequently, the same measures for identifying heterogeneity (cloning, mixing of multiple PCR reactions, see also below) can be applied for use in DNA barcoding. Nonetheless, addressing the problem of heterogeneity in the ITS2 clearly burdens the approach with additional time and expense. However, it is our contention that this extra burden is overshadowed by the significant savings in time and effort through the use of the automated analytical pipeline. No other phylogenetics marker or DNA barcoding candidate is similarly equipped for analytical high-throughput. Furthermore, no other potential barcode exhibits the same level of universality (i.e., in primers for PCR) than the ITS2. Thus, the ITS2 meets criterion one of the recommendations for a standard plant barcode [50]. Furthermore, our current assessment of primary and secondary sequence structure among an exhaustive survey of chlorophytan diversity indicates that ITS2 also meets Criteria Two (bi-directional sequencing with few or no ambiguities) and Three (enables the most species to be distinguished) of the CBOL recommendations [50].

With some notable exceptions [16], [51], [95], [102], the ITS2 gene has largely been shunned by those investigators that are designing or promoting DNA barcodes for the land plants [15], [50], [103], [104]. Concern about the confounding impact of pseudogenes and the potential presence of intraspecific or intra-individual variation (due to differing rates of homogenization of the rDNA tandem array or due to introgression) were cited as reasons for relegating ITS2 to, at best, a supporting role in DNA barcoding for the land plants [15], [50], [103]. The confounding influence of pseudogenes (from the aberrant secondary structures produced by ITS2 pseudogenes that have accumulated a substantive number of indels as a consequence of the loss of function of the ITS2 gene) can be minimized or eliminated by the use of DMSO during the PCR [104]. In addition, testing for the presence of conserved 5.8S rRNA motifs may be a relatively easy (i.e., amplifying the spacer region to include the 5.8S rRNA adds very little time and investment to an investigation of the ITS2) means of recognizing spacer pseudogenes [105]. At present, there have been no reports of ITS2 pseudogenes in the Chlorophyta, but this is likely to change as more chlorophytan taxa are scrutinized.

As was noted above, the issue of heterogeneity within a species or within an individual has the potential to be more problematic than the confounding issue of ITS2 pseudogenes. Regardless of the source, ITS2 heterogeneity has been deemed a liability for its use as a DNA barcode for the land plants [15], [103]. However, life history differences between most Chlorophyta and the embryophytes may account, at least in part, for the antipathy towards the ITS2. Specifically, many Chlorophyta exhibit zygotic meiosis and, thus, are vegetatively haploid. All embryophytes exhibit sporic meiosis and, thus, are vegetatively diploid. Therefore, the ITS2 in many Chlorophyta behaves more like an organellar gene that exhibits uniparental inheritance. Angiosperms will have two copies from each parent, thus doubling the opportunities for introducing heterogeneity. Introgression, which may play a role in the evolutionary history of a significant number of angiosperm taxa, is often cited as the culprit in producing multiple ITS alleles which, in turn, would likely confound a phylogenetic analysis [103], [104]. Except for some marine macrophytes that may exhibit sporic meiosis [106], [107], [108], [109], there seems to be little evidence of introgression [110] that could produce ITS2 heterogeneity in the Chlorophyta. Moreover, the positive results from the most recent and extensive investigations of ITS2 as a DNA barcode for plants [51], [95] suggest that the concerns regarding ITS2 may be overstated.

Lastly, we confront the issue of pragmatism. Although their work did not specifically address a DNA barcoding approach, Feliner and Rosseló [101] persuasively argue in favor of a multi-locus line of attack if ITS2 is to be used for assessing organismal diversity. However, as we stated in the Introduction, virtually all of the other candidate genomic targets for DNA barcoding in the Chlorophyta exhibit one or more serious deficiencies. The *rbc*L gene may be able to play a role in DNA barcoding for select groups (e.g., the Chlamydomonadales), but a lack of universal primers coupled with difficult or intractable chlorophytan groups compromises a taxonomically broad use of *rbc*L for the near term. At present, the ITS2 gene is the only viable candidate for immediate use in DNA barcoding for the Chlorophyta. Despite objections to the routine use of ITS2 for land plants, our tests of the ITS2 data demonstrate that this marker resolves major green algal lineages (some with high bootstrap support). Most importantly, our results dramatically illustrate that ITS2 data from unknown chlorophytan organisms can be plugged into a high resolution tool for taxonomic assessment. If the ITS2 gene can serve as a powerful DNA barcode, then this approach has the potential to help address some of the most complex problems in microbial ecology and diversity including analyses of community structure, the paradox of plankton, issues of dispersal and the nature or existence of biogeographical patterns among algal microbes.

## Materials and Methods

All phylogenetic analyses followed the procedure outlined in Schultz and Wolf (2009). Data were obtained (2009/09/30) from the ITS2 Database [65], [73], [111]. A global, multiple sequence-structure alignment of all available (591) chlorophycean ITS2 sequences with available secondary structures was generated in 4SALE v1.5 [70], [112]. Sequences and secondary structures were synchronously aligned, making use of an ITS2 sequence-structure specific scoring matrix [70], [112]. Accordingly, alignments were calculated for the Ulvophyceae (938 sequences) and Trebouxiophyceae (741 sequences). Further, a global Chorophyta tree was calculated that includes all the sequences described above for the individual class-specific trees. For each of the alignments, a set of all *Micromonas* (Prasinophyceae) sequences available in the ITS2 database was used as the outgroup. Based on primary and secondary structure information, phylogenetic relationships were reconstructed by Profile Neighbor-Joining (PNJ) [72], through the use of an ITS2 sequence-structure-specific, General Time Reversible (GTR) substitution model, in ProfDistS v0.9.8 [71], [74], [75]. In addition to the usual Windows/Mac/Linux GUIs, all of the methods described above may be used from a UNIX command line shell and thus be incorporated in any type of automated scripts. The complete procedure of data acquisition, alignment calculation and tree reconstruction took less than one hour of computational time for the three class-specific trees and 3.5 h for the complete Chlorophyta tree on a conventional 2.0 GHz single core computer.

In a second manual step we obtained bootstrap support values (Felsenstein, 1985) for the major taxonomic clades within the trees. For this step, manual profiles were set in ProfDistS with the Cartoon2Profile tool (http://profdist.bioapps.biozentrum.uni-wuerzburg.de/cgi-bin/index.php?section=cart2prof), after rooting and visualizing the distance trees with FigTree v1.2.3 [113]. Cartoon2Profile is a Perl script that converts cartoons as set in FigTree into a ProfDistS compatible profile file. Cartoon2Profile has been explicitly developed for this study, but may be used for any investigation that uses FigTree and ProfDistS. Calculation of bootstrap values with these profiles required less than 10 minutes of computational time using a desktop computer. We visualized a concatenated topology of the three class-specific trees in a hyperbolic tree based on the HyperGeny tree browser (http://bioinformatics.psb.ugent.be/hypergeny). The hyperbolic tree is publicly available as a supplement to this study at the ITS2-Database Supplements Page and at http://hypertree.bioapps.biozentrum.uni-wuerzburg.de.

At the present time, we are aware of no sequence-structure approach using individual secondary structures that can accommodate treeing methods other than the algorithmic approach of NJ. However, in order to provide an alternative context in which to evaluate the sequence-structure trees, a second set of analyses of nucleotide data only for each of the three classes of green algae and a composite analysis for the Chlorophyta was completed. These analyses employed an approximately maximum likelihood approach (ML) using FastTree 2 [114] with default settings. The sequence alignment was determined using Clustal [115].

## Supporting Information

File S1 Phylogenetic tree (in Newick format) from ML analysis (using FastTree 2) of sequence data only from the same set of chlorophycean taxa used in the sequence-structure analysis. This file is best viewed using FigTree (http://tree.bio.ed.ac.uk/software/figtree/).
(TREE)

File S2 Phylogenetic tree (in Newick format) from ML analysis (using FastTree 2) of sequence data only from the same set of trebouxiophycean taxa used in the sequence-structure analysis. This file is best viewed using FigTree (http://tree.bio.ed.ac.uk/software/figtree/).
(TREE)

File S3 Phylogenetic tree (in Newick format) from ML analysis (using FastTree 2) of sequence data only from the same set of ulvophycean taxa used in the sequence-structure analysis. This file is best viewed using FigTree (http://tree.bio.ed.ac.uk/software/figtree/).
(TREE)

File S4 Phylogenetic tree (in Newick format) from ML analysis (using FastTree 2) of sequence data only from the same set of chlorophytan taxa used in the sequence-structure analysis. This file is best viewed using FigTree (http://tree.bio.ed.ac.uk/software/figtree/).
(TREE)
